# Racial and Gender Discrimination When Tailoring Medical Management to Hypertension Treatment in Latin America: As a Key Player in the field, the Inter-American Society of Hypertension advocates equitable healthcare approaches in Latin America

**DOI:** 10.1093/ajh/hpaf050

**Published:** 2025-04-01

**Authors:** Luis Alcocer, Ernesto L Schiffrin, Gregory Fink, Mariela M Gironacci, María Claudia Irigoyen, Ana C Palei, Minolfa Prieto, Henry Punzi, Dora Inés Molina de Salazar, Carlos I Ponte-Negretti, Jose Ortellado, Ernesto Peñaherrera, Daniel Piskorz, Martin Rosas, Osiris Valdez, Raúl Villar, Fernando Wyss, Carlos M Ferrario

**Affiliations:** Instituto Mexicano de Salud Cardiovascular, Ciudad de México, México; Department of Medicine, Sir Mortimer B. Davis-Jewish General Hospital, McGill University, Montreal, Québec, Canada; Department of Pharmacology & Toxicology, Michigan State University, East Lansing, Michigan, USA; Department of Biological Chemistry, Faculty of Pharmacy and Biochemistry, University of Buenos Aires, Buenos Aires, Argentina; Laboratório de Análises Clínicas, Instituto do Coracao (InCor), Hospital das Clinicas HCFMUSP, Faculdade de Medicina, Universidade de Sao Paulo, São Paulo, Brazil; Department of Surgery, University of Mississippi Medical Center, Jackson, Mississippi, USA; Department of Physiology, Tulane University, New Orleans, Louisiana, USA; Punzi Medical Center, Carrollton, Texas, USA; Faculty of Health Sciences, University of Caldas, Manizales, Colombia; Caribbean Cardiologic Unit, Venezuelan Foundation of Preventive Cardiology, Caracas, Venezuela; Administration, Universidad Católica de Asunción, Universidad Uninorte, Asunción, Paraguay; Department of Cardiology, Hospital Luis Vernaza, Guayaquil, Ecuador; Rosario British Sanatorio, Rosario, Argentina; Department of Clinical Cardiology, Instituto Nacional de Cardiología, México City, México; Clinical Cardiology, Centro de Especialidades Médicas Romana (Cemer Clinic), La Romana, República Dominicana; Internal Medicine-Diabetology, IntegraMédica La Serena, Coquimbo, Chile; Unidad de Cardiologia, Cardiovascular Services and Technology of Guatemala, Guatemala City, Guatemala; Department of Surgery, Wake Forest School of Medicine, Winston Salem, North Carolina, USA

**Keywords:** blood pressure, cardiovascular disease, health care, hypertension, Latin America, stroke, women’s health

## Abstract

Discrimination in cardiovascular healthcare, particularly concerning hypertension treatment, is a significant and complex issue in Latin America, driven by biases related to gender, ethnicity, and economic status. Although cardiovascular disease is the leading cause of death worldwide, disparities in healthcare delivery endure, especially impacting marginalized populations. Women, ethnic minorities, and economically disadvantaged groups encounter considerable barriers, including underrepresentation in clinical research, delayed diagnoses, and unequal access to guideline-recommended treatments. Economic disparities maintain a divided healthcare system in which the quality of treatment often directly correlates with socioeconomic status, reinforcing inequities and adversely affecting health outcomes in lower-income communities. Ethnic discrimination, stemming from deeply ingrained social biases, leads to inadequate care and limited access to advanced medical technologies, disproportionately impacting indigenous and Afro-descendant populations. Addressing these systemic inequities requires comprehensive strategies that ensure equitable participation in clinical trials, develop tailored public health policies sensitive to socioeconomic and cultural contexts, and implement targeted educational initiatives. Healthcare systems must actively dismantle entrenched biases, improve access for economically disadvantaged communities, and guarantee that ethnic minorities receive treatment of equal quality. The Inter-American Society of Hypertension emphasizes that removing these discriminatory barriers reduces the burden of cardiovascular disease and enhances overall health outcomes across Latin America. This document endorses consensus recommendations detailed in positions 1 through 4, which tackle specific challenges related to personalized care, racial biases in treatment algorithms, socioeconomic healthcare inequalities, and gender disparities in hypertension management.

## Graphical Abstract

**Graphical Abstract:**
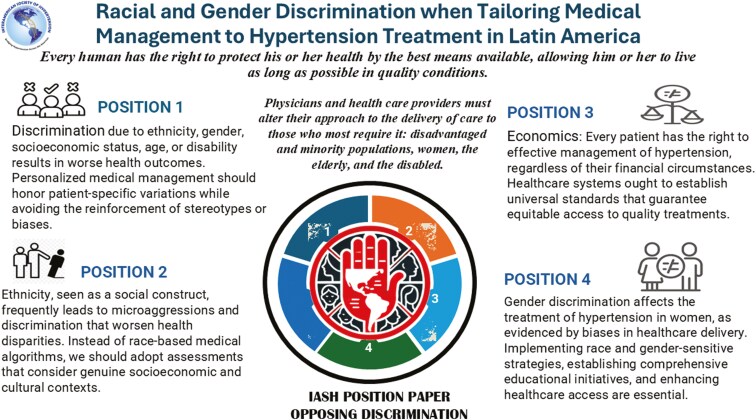

Latin America’s (LA) diversity is one of its defining features in the American continent, with each country embodying unique combinations of race, ethnicity, history, language, and economics.^1,2^ Various indigenous, European, African, and Asian populations determine profound cultural and political influences on the region. The diversity of LA is rooted in its historical, racial, and economic complexities. Its languages, cultures, and identities are the products of centuries of blending, resistance, and adaptation, with ongoing efforts to address inequalities and celebrate its complex heritage.^3,4^

In LA, race, ethnicity, culture, and religion have a marked influence on the global burden of infectious diseases and health. Bolivia, Guatemala, and Peru have a significant proportion of indigenous populations. In contrast, populations in Argentina, Uruguay, and Chile are predominantly of European descent, following the large-scale European immigration in the 19th and 20th centuries.^3^ Cultural and demographic influences, partly due to the past African slave trade, imprint health delivery in Brazil, Cuba, the Dominican Republic, and areas of Colombia and Venezuela. Many countries, including Mexico, Honduras, and EL Salvador, have predominantly mestizo populations, reflecting a mix of indigenous and European ancestry. On the other hand, countries like Peru and Brazil have a significant presence of Asians (Japanese, Chinese, and Korean), contributing to cultural and social diversity. In LA, the diversity of racial and cultural influences parallels the varied economic development,^5^ with Chile, Uruguay, and Panama possessing stable economies and higher human development indexes. In contrast, countries like Haiti, the Dominican Republic, Honduras, and Nicaragua struggle with poverty, limited infrastructure, and reliance on agriculture. Across most of LA, income inequality remains a significant issue, deeply tied to past colonial land distribution, systemic racism, and limited access to comprehensive health care.

Socioeconomic inequality, geographic diversity, cultural factors, and race disparities affect access to services, quality of care, and health outcomes across socioeconomic classes, ethnic groups, rural versus urban populations, and genders. Health systems in LA often lack gender-sensitive policies, leading to unequal treatment.^6^ Cultural norms and “machismo” discourage men from seeking care and reinforce traditional caregiving roles for women, limiting their opportunities to focus on their health.

Cardiovascular disease (CVD) is LA’s leading cause of death, reflecting the region’s epidemiological transition from infectious diseases to chronic noncommunicable diseases.^7,8^ Urbanization, lifestyle changes, and aging populations drive this shift. The shift to higher cardiovascular death rates is not a universal phenomenon in LA, as a significant decrease in CVD mortality over communicable diseases was demonstrated in Argentina, Chile, Brazil, and Colombia.^7^

The magnitude of CVD and the cardio-renal-metabolic syndrome in LA has been recently documented in the 2024 Global Burden of Disease study, a comprehensive effort of the WHO to quantify health loss across places and over time.^9,10^ In LA, studies confirm that men generally have higher blood pressure and a higher prevalence of hypertension compared to women.^11,12^ However, this disparity is age-dependent, with women having a higher prevalence in older age groups. In concordance with studies obtained on Hispanic women in the USA, the limited data on gender differences in LA demonstrate a higher prevalence of hypertension in women.^13–16^ A 44% hypertension prevalence (self-reported blood pressure > 140/90 mm Hg) in urban and rural communities in Argentina, Brazil, Chile, Colombia, Peru, and Uruguay was reported in a cross-sectional study.^17^ A survey of 7,524 adults (aged 35–74 years) in the Southern Cone of LA found that a significantly higher percentage of treated hypertensive women (62.1% vs. 36.1% of men) failed to achieve blood pressure control.^18^ The SALURBAL (**Sal**ud **Urb**ana en **A**merica **L**atina/Urban Health in LA) project carried out on 109,184 adults (aged 18–97 years) from 230 LA cities demonstrated that lower odds of hypertension in women were associated with university education.^19^ These findings agree with the report that educational attainment, more than income or occupation, strongly predicts hypertension prevalence in a recent meta-analysis of 51 studies in LA.^20^

We have used the recent data published by Mensah *et al*.^21^ from the Global Burden of Cardiovascular Disease Initiative^22^ to emphasize how cultural and socioeconomic differences among the diverse geographic subregions regions of LA influence the magnitude of the CVD burden when compared with the North American countries of the United States and Canada. **Figure 1** shows the prevalence rates of ischemic heart disease and stroke, [calculated as the ratio of the number of cases (people with the condition) divided by the total size of the population], in the basic geographical subregions of LA [Southern LA (Argentina, Chile, Uruguay), Andean western countries (Bolivia, Ecuador, Peru), Central LA (Colombia, Costa Rica, El Salvador, Guatemala, Honduras, Mexico, Nicaragua, Panama, Venezuela), and Tropical LA (Brazil, Paraguay)]. The prevalence rates of ischemic heart disease and strokes are proportionally similar in the countries comprising the regions of Southern and Central LA, with lower prevalence rates documented in countries included in the Andean and Tropical LA. **Figure 2** shows that hypertension prevalence rates in Tropical and Andean LA countries are essentially the same as those calculated for the pooled High-Income North American countries. These data underscore the high sensitivity of Latin American residents to the development of cardio-renal-metabolic disease.

**Figure 1.**
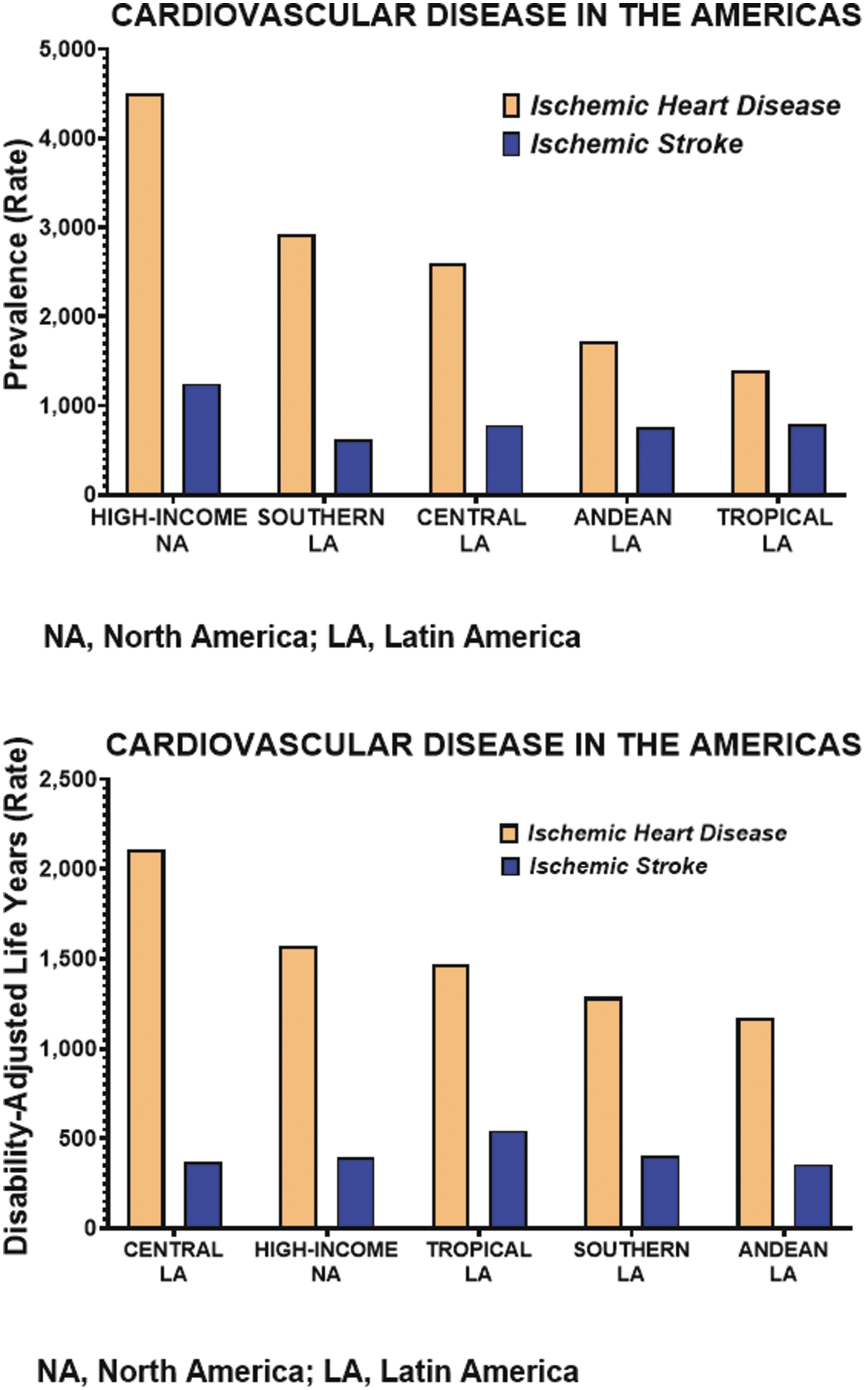
Comparative prevalence and DALY for ischemic heart disease and ischemic stroke in Latin American subregions compared to the North America composite of Canada and the USA. The prevalence of ischemic heart disease within the LA subregions is highest in the composite of Argentina, Chile, and Uruguay (Souther LA) and Central LA (Colombia, Costa Rica, El Salvador, Guatemala, Honduras, Mexico, Nicaragua, Panama, Venezuela). On the other hand, Central LA exhibits the highest rate of DALY, reflecting the health stresses imposed by socioeconomic factors. The prevalence rate is calculated using the Disease Model—Bayesian meta-regression (DisMod-MR 2.1).^23,24^ The DALY rate expresses years of life lost due to premature death and years lived with a disability of specified severity and duration.^25^ The graph was generated from numerical data reported by Mensah *et al*.^21^

**Figure 2.**
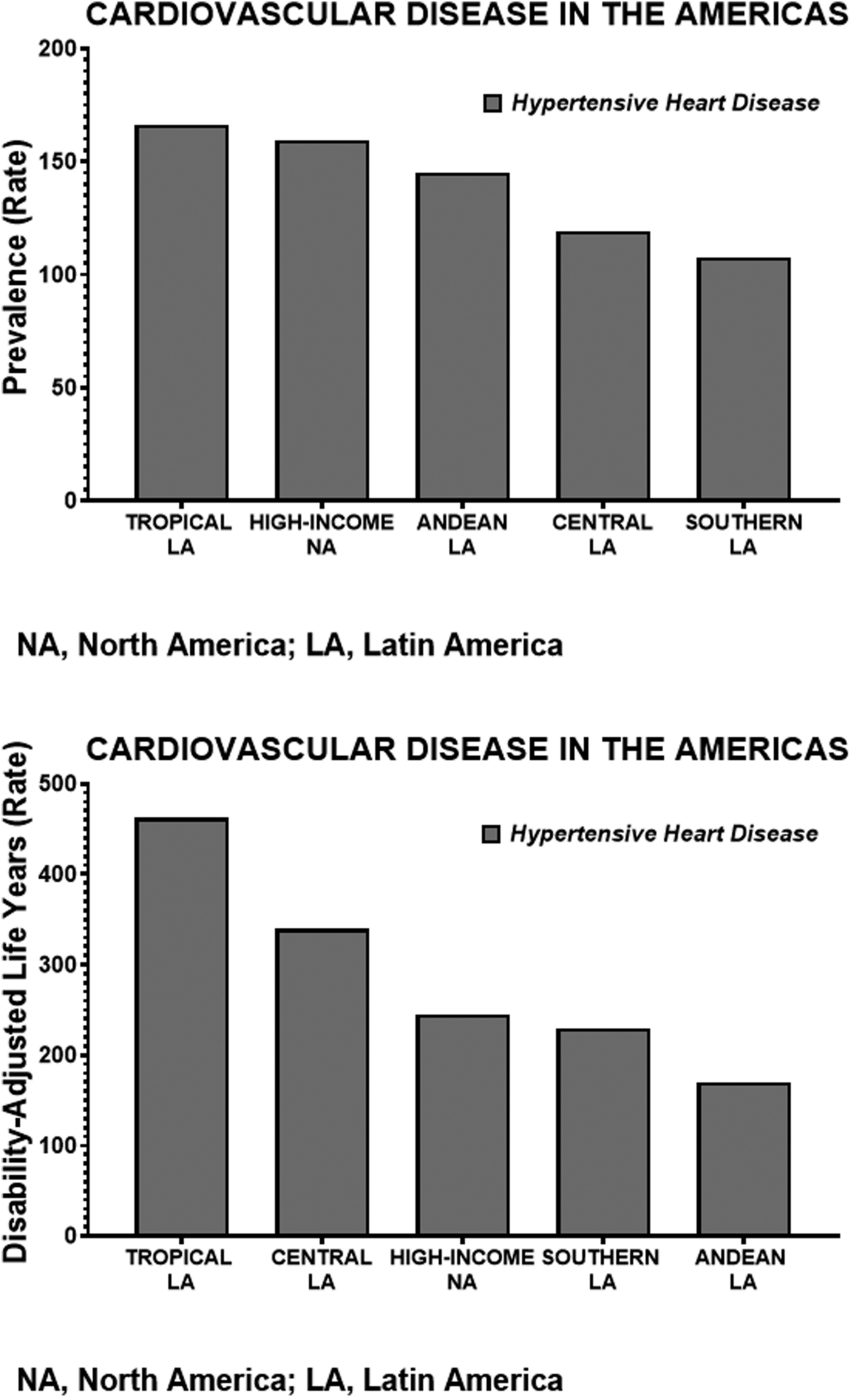
Comparative prevalence rate and DALY for hypertensive heart disease in Latin American subregions compared to the North America composite of Canada and the USA. Other abbreviations and procedure approaches are defined in Figure 1 legend.

Disparities in the treatment of hypertension based on race, gender, age, and socioeconomic status (SES) remain poorly recognized in LA.^26^ These factors can influence access to care, adherence to treatment, and even the type of interventions provided. Addressing disparities in hypertension treatment requires tailoring medical management to consider these factors. Improved access to care, better education on hypertension management, and individualized treatment based on gender, age, and socioeconomic context are critical in overcoming these barriers. Ethnic, cultural, nutritional, and genetic imprints in the Latin American culture impose a heavy burden through increased rates of hypertension, metabolic syndrome, type 2 diabetes, heart failure, and chronic kidney disease. As reviewed elsewhere,^27^ the INTERHEART^28^ and INTERSTROKE^29^ landmark cardiovascular risk factor studies underscored the influence of abdominal obesity as the highest attributable population risk for myocardial infarction, ischemic and hemorrhagic stroke, respectively.

Healthcare delivery is rife with entrenched and systemic gender inequities in LA. Gender discrimination in LA is a real and dangerous problem affecting women’s health and well-being. The combined effect of access to care, health insurance, and medical care affordability has a predominant influence on the delivery of women’s care. Although women’s health discrimination is relatively universal, the burden imposed by patriarchal cultural norms and religious and socioeconomic factors in LA impacts the provision of care to women.^30^ A study examining adults in 230 Latin American cities found that the association between socioeconomic status and hypertension varied by gender.^19^ This observation highlights the importance of context- and gender-sensitive approaches to reduce regional hypertension prevalence. Although precise documentation of women’s discrimination in healthcare access and treatment is challenging to find, a survey by KFF, an independent source for health policy research, documented that 21% of Hispanic, low-income, uninsured women (Ages 18–64 years) have difficulties in accessing care.^31^ Excess female mortality across the life cycle is a corollary of gender-related power imbalances in LA. In addition, prejudicial cultural norms and stereotypes also account for a thick glass ceiling that limits transformative policies geared to advance opportunities in education and work for women.^32^

This article summarizes a Consensus Opinion assembled by the officers of the Inter-American Society of Hypertension (IASH) and invited opinion experts during the 2024 International Society of Hypertension Biennial Meeting (Cartagena, Colombia, 19–22 September 2024) on the negative impact of discriminatory factors in the Latin American population at risk for hypertension and cardio-renal-metabolic disease. The following publications^19,32–37^ may be perused by readers wishing to delve further into these issues. The IASH, a pioneered assembly of physicians, basic scientists, and public health specialists, spearheaded the development of LA Hypertension Societies since its founding at a satellite symposium of the 1974 World Congress of Cardiology in Mendoza, Argentina.^38^

## CONSENSUS RECOMMENDATIONS

### Position 1:

***As stated by Richard Allen Williams,*** **^39^** ***we affirm that every human has the right to protect their health by the best means available, allowing them to live as long as possible in quality conditions. We stress that physicians and health care providers must alter their approach to the delivery of care to those who most require it: disadvantaged and minority populations, women, older people, and people with disabilities.***

IASH recognizes that gender, age, and socioeconomic conditions of the persons living with hypertension are factors that result in unique conditions that must be considered for the appropriate personalized management of elevated blood pressure. However, when these characteristics are used directly or indirectly as an identity label for a person and a justification to deny, condition, or reduce access to quality management, it constitutes an act of discrimination (i.e., the unjust or prejudicial treatment of different categories of people, especially on the grounds of ethnicity, ancestry, age, sex, or disability).

Every human has the right to protect their health by the best means available, allowing them to live as long as possible with a good quality of life. We understand discrimination in the management of high blood pressure as any recommendation or decision that results in a decrease in the quality of medical care provided based on preconceived notions about the identity of an individual or group and that this is a widespread problem with significant social, psychological, and economic repercussions. While all medical management must be personalized, individualizing care requires recognizing differences. These differences must be analyzed using an individual vision, not in a generic way.

Although medical practice has improved markedly with evidence-based medicine, personalized approaches are still far from perfect and far from precision medicine, in which each person receives the management they require according to their genetic, behavioral, socioeconomic, and environmental status.

Two different approaches to personalized treatment influence the discriminatory application of health resources to the general population. The first approach considers age, gender, ethnicity, or socioeconomic condition to capture variants influencing patient management. The second approach is to use these profiles as constituents of stereotyped prejudices toward older people, Afro-descendant, Indigenous origin, obesity, or social and cultural ancestral ties to the lands and natural resources where they live, occupy, or from which they have been displaced. This type of discrimination, deeply rooted in LA’s history, results in patients from minorities being denied more advanced or more expensive medical technologies. This type of discrimination is often rooted in the provider’s or decision-maker’s unconscious biases in health^40–42^ and financial restraints. It is suggested that implementing precision medicine, supported by artificial intelligence, will ensure that each person’s individuality is determined by almost all the traits that make it up.

### Position 2:

***Treatment algorithms structured by race are based on inaccurate historical beliefs, positing that people of African descent are physiologically different according to their ethnicity.*** **^39,43–49^** ***This criterion should be modified by algorithms adjusted by the socioeconomic and cultural conditions in which the patient lives and which are conditioned by their ethnic classification, without justifying lower quality management.***

Race, as a word, has its roots in the Latin *generatio* (a begetting).^50^ Racism is a social construct that emphasizes the racial phenotype as an indicator of the quality of the person and innate inferiority that is the reason for macroaggressions.^51^ Ethnic discrimination in health care is a widespread problem affecting the quality of care received by minority populations.^42^ In medicine, ethnicity generates microaggression-related discrimination, often unconscious, which justifies a different treatment, often of inferior quality, and justified by apparent biological differences conditioned by race.

Although race or ethnicity are factors associated with higher rates of illness, access to health care, and morbidity and mortality,^52^ they are not valid biological determinants of genetic factors of hypertension since racial definitions are primarily social in origin rather than genetic causes of disease.^53^ As documented by Williams *et al*.,^52^ ethnicity is a marker of different socioeconomic situations, which can be due, at least in part, to different availability and access to healthcare modalities. Barriers to accessing high-quality antihypertensive treatment have significant implications for health outcomes among ethnic minorities. These populations are disproportionately affected by increased risk of uncontrolled hypertension, leading to higher rates of cardiovascular events and all-cause mortality.^54^ Treatment disparities also contribute to the overall health inequalities found in these communities. Traditional algorithms, adjusted for phenotypes based on t gender, race, or ethnicity, are manifestations of structural discrimination and must be replaced *by algorithms based on actual biological and socioeconomic differences.* These considerations do not negate the existence of phenotype differences in salt sensitivity and blunted blood pressure response to renin-angiotensin system inhibitors in blacks.^49,55^ The CREOLE study^56^ showed a better blood pressure response to the combination of a diuretic and calcium channel blocker in African women compared with African men. Racial differences in salt sensitivity may be a corollary of lower potassium intake.^57^

### Position 3:

***Every patient with hypertension has the human right to receive optimal treatment for the rest of their life. So-called “basic” treatment should be considered an economic strategy in public health of a palliative nature and only of transitory benefit. The perpetuation of basic treatment for an individual or group, dictated by socioeconomic factors, constitutes a discriminatory strategy. IASH endorses all activities and actions that allow universal and timely access to optimal management.***

The categorization of antihypertensive treatment into two conditions, Basic and Optimal, justifies and promotes a different way of treating hypertension, depending on people’s opportunities for economic and social access to health care. This approach to hypertension treatment addresses the challenge of limited access to quality healthcare, a common issue in communities, countries, and regions with unfavorable socioeconomic conditions. However, it also perpetuates a system of stratified healthcare delivery, institutionalizing inequality under the guise of resource constraints. Current clinical practice guidelines are slowly adapting to underscore sex differences in underlying mechanisms of hypertension and cardiovascular disease. Nobakht *et al*.^58^ have called attention to the distinct elements of hypertension management in women and the need to consider a multidisciplinary team-based approach that focuses on woman’s life cycle, underlying medical conditions, socioeconomic stressors, and access to care.

Cardiovascular disease in LA inflicts substantial financial costs and loss of productivity that translates into a high number of disability-adjusted life years (DALYs) lost (**Figure 1**). A 2022 report by the Fifarma organization (https://fifarma.org) documented that treatment of high mortality diseases (neoplasms and cardiovascular diseases) cost Argentina, Brazil, Mexico, Colombia, Chile, Ecuador, Costa Rica, and Peru 196.5 billion dollars.^33^ These data agree with the cardiovascular death estimates generated by the 2019 Global Burden of Disease Study 2019.^59^

The so-called basic management, to replace the optimal treatment, is a primarily intuitive strategy of cost containment based on acquisition costs and some cost-effectiveness studies but not really on a cost-utility analysis that ensures that the person who receives it will have a benefit in quality of life and time like or not less than that obtained with the optimal treatment. This simple treatment strategy can legitimize inequality for economic reasons. (One type of medicine is for low-income people, and another is for the nonpoor.)

### Position 4:

***Gender discrimination in the medical management of hypertension in women is a multifaceted problem that requires urgent attention.***

Until recently, reproductive and gynecologic health predominated resources associated with women’s medical care. Ensuring equitable representation of women in clinical trials is crucial for developing effective and safe medical treatments. Historically, women, particularly women of color, have been underrepresented in clinical research, leading to health inequities and social injustice. This underrepresentation limits our understanding of sex-specific responses to treatments and contributes to disparities in healthcare outcomes.

Health systems must adopt gender-sensitive approaches to address gender disparities in treating hypertension.^60^ This includes increasing awareness among healthcare providers about gender biases and developing gender-specific guidelines for hypertension management. Empowering women through education and improving access to healthcare services are crucial to achieving better health outcomes.

Gender discrimination in health care can lead to disparities in diagnosis, treatment, and outcomes, requiring a thorough examination of these issues. While both men and women are susceptible to developing hypertension, research shows that women often face unique challenges in their treatment. Psychosocial factors, such as depression and mood disorders, together with the burden of childcare and family care, can contribute to late diagnosis and inadequate adherence to treatment, increasing the risk of CVD in women more than in men. Comprehensively implementing these strategies can foster greater equality of opportunity and participation.

Biological and physiological differences between men and women contribute to variations in the prevalence and progression of hypertension. Women, especially postmenopausal women, experience a sharper increase in blood pressure starting from the third decade of life, leading to a convergence in hypertension prevalence with men as they age.^11^ Sex-specific factors, such as more vascular and myocardial stiffness, influence suboptimal treatment outcomes in women.^12^ These differences are often overlooked in clinical practice, leading to suboptimal management of hypertension in women. Socioeconomic factors further exacerbate gender disparities in hypertension management. Women, particularly those from marginalized communities, may face barriers such as limited access to health care, financial constraints, and lack of health literacy. These factors hinder women’s ability to seek timely medical attention and adherence to treatment plans.

The consequences of gender discrimination in the management of hypertension are profound. Women with poorly controlled hypertension are at increased risk of developing severe cardiovascular complications, which can significantly affect their quality of life and increase mortality rates. In addition, the psychological burden of sex discrimination can lead to increased stress and anxiety, further aggravating hypertension. Health systems must adopt gender-sensitive approaches to address gender disparities in the treatment of hypertension. This approach should include increasing awareness among healthcare providers about gender biases, ensuring equitable representation of women in clinical trials (see below), and developing gender-specific guidelines for hypertension management. In addition, empowering women through education and improving access to healthcare services are crucial to achieving better health outcomes.

IASH endorses the following strategies to enhance the inclusion of women in clinical trials:

1. *Policy Implementation:* Mandating the inclusion of women in clinical research is essential. For instance, the US National Institutes of Health (NIH) Revitalization Act of 1993 (https://openlibrary.org/books/OL14687929M/National_Institutes_of_Health_Revitalization_Act_of_1993) requires women and minorities to be included in National Institutes of Health-funded research.
2. *Addressing Barriers to Participation:* Identifying and mitigating obstacles that prevent women from participating in clinical trials can improve enrollment. Barriers may include lack of awareness, caregiving responsibilities, and socioeconomic factors. Practical interventions, such as childcare and transportation, flexible scheduling, and community engagement, can facilitate participation.^61^
3. *Comprehensive Reporting and Analysis:* It is vital to ensure that clinical trials include women and analyze and report data by sex. This practice helps identify sex-specific effects of interventions, leading to more personalized and effective treatments.^62^
4. *Educational Initiatives:* Training researchers and healthcare professionals on the importance of sex and gender considerations in clinical research can promote more inclusive study designs and analyses.^63^ We endorse any activity and recommendations to reverse gender inequity in health system management and perpetuate women’s underrepresentation in medical leadership.^32^
5. *Implementing Public Health Strategies:* Initiatives incorporating the combination of drugs as a simple pill, community-based interventions based on pharmacist-based management, and educative activities at barbershops and beauty salons effectively address health inequalities (see Vaduganathan *et al*.^22^ for a comprehensive discussion of these activities).

Implementing these strategies can lead to more equitable representation of women in clinical trials, improve health outcomes, and advance social justice.

Discrimination in cardiovascular healthcare, particularly regarding hypertension treatment, significantly impacts marginalized populations in LA, driven by gender, ethnic, and economic biases. These biases contribute to pronounced disparities in healthcare access, diagnosis, treatment, and outcomes, disproportionately affecting women, ethnic minorities, and economically disadvantaged groups.

Despite CVD (CVD) being the leading cause of death in women worldwide, women are often underrepresented in clinical research, overlooked in medical education, and subjected to biases in healthcare delivery.^64,65^ These issues are markedly amplified in LA due to historical and religious biases. This gap limits understanding of how CVD presents and progresses differently in women, as well as how they respond to treatments compared to men. Social determinants of health, such as income inequality, caregiving responsibilities, and limited access to healthcare, disproportionately affect women. These factors compound their challenges in managing cardiovascular risk factors and accessing timely care. Medical education and public health campaigns have historically emphasized men’s cardiovascular health, leaving women under-informed about their risks. For example, many women remain unaware that CVD is their leading health threat, mistakenly perceiving breast cancer as the most significant risk.

Economic discrimination institutionalizes a tiered healthcare system, limiting optimal hypertension management for poorer communities. Ethnic discrimination, rooted in social prejudices, reduces the quality of care available to Indigenous and Afro-descendant populations. Addressing these multifaceted forms of discrimination requires systemic interventions, including equitable representation in clinical trials, implementation of culturally and economically sensitive public health policies, targeted educational programs, and awareness campaigns. Active dismantling of entrenched biases within healthcare systems is critical for improving cardiovascular outcomes and achieving equitable healthcare across LA. Medical Societies, health policymakers, and providers should be empowered to address these issues.^66^ The positions outlined by the IASH offer practical strategies for combating discrimination and advancing comprehensive, fair, and inclusive hypertension management.
